# Molecular mechanisms of the microRNA-132 during tumor progressions

**DOI:** 10.1186/s12935-021-02149-7

**Published:** 2021-08-21

**Authors:** Meysam Moghbeli, Amir Sadra Zangouei, Zahra Nasrpour Navaii, Negin Taghehchian

**Affiliations:** 1 Department of Medical Genetics and Molecular Medicine, School of Medicine, Mashhad University of Medical Sciences, Mashhad, Iran; 2 Student Research Committee, Faculty of Medicine, Mashhad University of Medical Sciences, Mashhad, Iran; 3 Department of Chemistry, Faculty of Science, Ferdowsi University of Mashhad, Mashhad, Iran

**Keywords:** MiR-132, Cancer, Diagnosis, Prognosis, Marker

## Abstract

Cancer as one of the leading causes of human deaths has always been one of the main health challenges in the world. Despite recent advances in therapeutic and diagnostic methods, there is still a high mortality rate among cancer patients. Late diagnosis is one of the main reasons for the high ratio of cancer related deaths. Therefore, it is required to introduce novel early detection methods. Various molecular mechanisms are associated with the tumor progression and metastasis. MicroRNAs (miRNAs) are a class of non-coding RNAs (ncRNAs) family that has important functions in regulation of the cellular processes such as cell proliferation, apoptosis, and tumor progression. Moreover, they have higher stability in body fluids compared with mRNAs which can be introduced as non-invasive diagnostic markers in cancer patients. MiR-132 has important functions as tumor suppressor or oncogene in different cancers. In the present review, we have summarized all of the studies which have been reported the role of miR-132 during tumor progressions. We categorized the miR-132 target genes based on their cell and molecular functions. Although, it has been reported that the miR-132 mainly functions as a tumor suppressor, it has also oncogenic functions especially in pancreatic tumors. MiR-132 mainly exerts its roles during tumor progressions by regulation of the transcription factors and signaling pathways. Present review clarifies the tumor specific molecular mechanisms of miR-132 to introduce that as an efficient non-invasive diagnostic marker in various cancers.

## Background

Cancer is one of the main causes of human deaths worldwide, with an estimated 10.0 million deaths in 2020 [1]. It is the second leading cause of mortality in the United States with about 606,520 deaths in 2020 [2, 3]. The financial burden of cancer poses different challenges for the patients and healthcare system [4]. As the morphologically similar tumors may exhibit different clinical symptoms due to their molecular differences, it is of high importance to introduce non-invasive methods to assess the molecular differences in tumors to select the most efficient therapeutic option. As the non-protein-coding DNA covers almost 97% of the human genome, non-coding RNAs (ncRNAs) have become the frontier of cancer biology [5, 6]. They are categorized into the various families such as microRNAs (miRNAs), long noncoding RNAs (lncRNAs), small interfering RNAs (siRNAs), and circular RNA (circRNA) [7–9]. MiRNAs are a class of the short ncRNAs involved in post-transcriptional regulation through binding to 3 untranslated region (3-UTR) of the target mRNA that results in mRNA degradation or translational inhibition [10]. Considering, the crucial functions of miRNAs in regulation of cellular mechanisms including cell proliferation, differentiation, growth, and apoptosis [11, 12], aberrant miRNA expression can be correlated with various cancers [13]. MiRNAs may serve as tumor suppressors, oncogenes, and regulators of the self-renewal process in cancer stem cells (CSC) [14]. Dysregulated miRNAs are promising diagnostic tumor markers and are also efficient as novel targets for the cancer therapy [15]. Since, they have higher stability in body fluids compared with mRNAs, the expression profiling of circulating miRNAs in body fluids can be utilized as a non-invasive method for cancer diagnosis and prognosis [16–20]. MiR-132 is a critical regulator of various cellular processes such as angiogenesis, cell proliferation, migration, and apoptosis [21–23]. Aberrant expression of *miR-132* has been frequently reported in various cancers. It functions as a tumor suppressor or oncogene in different cancers [24–27]. Therefore, we have summarized all of the studies which have been reported the role of *miR-132* during tumor progressions. We categorized the *miR-132* target genes based on their cell and molecular functions (Table 1).

**Table 1:** Molecular targets of miR-132 during tumor progressions

Study	Year	Type	Gene	Target	Samples	MiR-132 function
Zhang et al. [23]	2014	Breast	miR-132	HN1	10 NT* NMuMG, 4T1, MDA-MB-231, and MCF10A cell lines	Tumor suppressor
Lian et al. [119]	2016	Laryngeal	miR-132	FOXO1	10 NT Hep-G2 and AMC-HN-8 cell lines	Oncogene
Han et al. [132]	2020	Retinoblastoma	ILF3-AS1	miR-132	50 NT Y79, HXO-RB44, SO-RB50, and RB1 cell lines	Tumor suppressor
Zhang et al. [129]	2020	Hepatocellular	LINC00160	miR-132	68 NT HCCLM3, Huh7, Hep3B, and MHCC97 cell lines	Tumor suppressor
Zhang et al. [123]	2019	Pancreatic	miR-132	PTEN	60 NT PAN-1, KLM-1, and PaCa-2 cell lines	Oncogene
Renjie et al. [45]	2015	Pituitary	miR-132	SOX5	16 T MMQ and GH3 cell lines	Tumor suppressor
Xie et al. [124]	2018	Breast	miR-132	PTEN	53 NT MCF-7 cell line	Oncogene
Chen et al. [94]	2016	Glioma	miR-132	TTK	46 T and 9 N U87 cell line	Tumor suppressor
Song et al. [140]	2017	Colorectal	XIST	miR-132	50 NT SW480, SW620, LOVO,HT29, and HCT116 cell lines	Tumor suppressor
Li et al. [68]	2016	Glioma	miR-132	SIRT1	U251 and U87 cell lines	Tumor suppressor
Zhao et al. [144]	2019	Pancreatic	miR-132	SHH	23 T and 25 N MiaPaCe-2a cell line	Oncogene
Zhang et al. [79]	2019	Ovarian	miR-132	BMI1	SKOV3	Tumor suppressor
Xue et al. [43]	2020	Nasopharyngeal	LINC01551	miR-132	24 NT HNE1, SUNE2, HONE1, CNE2, and 6-10B cell lines	Tumor suppressor
Liu et al. [89]	2018	Colorectal	MIAT	miR-132	30 NT Ht29, SW480, and LOVO cell lines	Tumor suppressor
Zhou et al. [51]	2018	Glioma	NEAT1	miR-132	14 T and 5 N U87, U251, SHG-44, and U-118MG cell lines	Tumor suppressor
Liu et al. [42]	2020	Pancreatic	PTTG3P	miR-132	60 NT AsPc-1, BxPC-3, CaPAN-2, MiaPaCa-2, PANC-1, and SW1990 cell lines	Oncogene
Chen et al. [36]	2019	Thyroid	miR-132	FOXA1	30 NT TPC1 and GLAG-66 cell lines	Tumor suppressor
Tian et al. [64]	2016	Ovarian	miR-132	E2F5	32 NT SKOV3, OVCAR3, and A2780 cell lines	Tumor suppressor
Guo et al. [84]	2018	Lung	miR-132	USP9X	A549 and NCI-1299 cell lines	Tumor suppressor
Wang et al. [35]	2018	Breast	miR-132	FOXA1	30 NT SK-BR3 and MDA-MB-468 cell lines	Tumor suppressor
Li et al. [128]	2019	Breast	miR-132	LAPTM4B	131 T and 87 N MCF-7, MCF-10A, ZR-75–1, T470, and MDA-MB-231 cell lines	Tumor suppressor
Lei et al. [147]	2015	Hepatocellular	miR-132	YAP	Huh7 and HepG2 cell lines	Tumor suppressor
Liu et al. [49]	2019	Bladder	CIRC-DOCK1	miR-132	23 T and 32 N BIU-87, EJ-m3, T24, and 5673 cell lines	Tumor suppressor
Qu et al. [108]	2016	Prostate	miR-132	GLUT1	PC-3 and DU-145 cell lines	Tumor suppressor
Geng et al. [112]	2016	Astrocytoma	miR-132	PEA15	U251 and U87 cell lines	Tumor suppressor
Chen et al. [126]	2018	Thyroid	miR-132	CSDE1	BCPAP, TPC1, and 8505c cell lines	Tumor suppressor
Cheng et al. [90]	2017	Glioblastoma	miR-132	TUSC3	U87MG	Oncogene
Liu et al. [58]	2015	Osteosarcoma	miR-132	SOX4	MG63, HOS, 143B, U2OS, and SaOS-2 cell lines	Tumor suppressor
Abukiwan et al. [136]	2019	Pancreatic	miR-132	TGFβ	35 NT AsPC-1 and PANC-1 cell lines	Oncogene
Zhao et al. [130]	2015	Cervical	miR-132	SMAD2	20 NT HeLa and C33A cell lines	Tumor suppressor
Li et al. [61]	2015	Lung	miR-132	SOX4	H460, A549, and YTMLC-9 cell lines	Tumor suppressor
Chen et al. [133]	2020	Oral	miR-132	TGFβ	37 NT SCC-9 and CAL-27 cell lines	Tumor suppressor
Zhang et al. [70]	2019	Colorectal	SNHG5	miR-132	25 NT RKO, SW480, and LOVO cell lines	Tumor suppressor
Li et al. [30]	2015	Gastric	miR-132	FOXO1	28 NT AGS and SNU-5 cell lines	Oncogene
Lin et al. [74]	2016	Ovarian	miR-132	CDH2, VIM	SKOV3 and OV2008 cell lines	Tumor suppressor
Liu et al. [139]	2019	Colorectal	miR-132	ERK1	NCM460, LOVO, and SW480 cell lines	Tumor suppressor
Liu et al. [102]	2017	Gastric	miR-132	CD44, FN1	201 NT BGC823, AGS, and HGC27 cell lines	Tumor suppressor
He et al. [107]	2017	Gastric	miR-132	MUC13	40 NT MKN28 cell line	Tumor suppressor
Huang et al. [62]	2020	Hepatocellular	miR-132	SOX4	HepG2, Huh7, and HccLM3 cell lines	Tumor suppressor
Liu et al. [78]	2017	Cervical	miR-132	BMI1	104 NT HeLa, SiHa, and C33A cell lines	Tumor suppressor
Zhang et al. [80]	2018	Lung	SOX2OT	miR-132	48 NT A549, H1299, NCI-H460, and HCC-827 cell lines	Tumor suppressor
Wei et al. [131]	2019	Bladder	miR-132	SMAD2	32 NT T24 cell line	Tumor suppressor
He et al. [86]	2020	Colorectal	SNHG16	miR-132	50 NT SW480 and SW620 cell lines	Tumor suppressor
Fu et al. [60]	2016	Prostate	miR-132	SOX4	57 NT LnCap and VCap cell lines	Tumor suppressor
Zheng et al. [81]	2014	Colorectal	miR-132	ZEB2	62 NT HT29, LOVO, HTC116, SW480, and SW620 cell lines	Tumor suppressor
Wang et al. [113]	2014	Osteosarcoma	miR-132	CCNE1	10 NT HOS, MG63, 143B, and Saos-2 cell lines	Tumor suppressor
Li et al. [32]	2016	Nasopharyngeal	miR-132	FOXA1	CNE2	Tumor suppressor

^*^ Tumor (T) tissues and Normal (N) margins

## Transcription factors

### Forkhead box proteins (Fox) transcription factors

There are increasing numbers of the feedback loop interactions between transcription factors and miRNAs in which the transcription factors up or down regulate the miRNAs, while the miRNAs inhibit the transcription factors in a negative feedback [28]. FOXO1 belongs to the Forkhead box proteins (Fox) transcription factors that functions as a negative regulator of cell cycle progression [29]. It has been shown that *miR-132* significantly promoted gastric tumor cell growth by FOXO1 targeting. There was also significant *miR-132* up regulation in gastric cancer (GC) tissues in comparison with normal margins [30]. Forkhead box protein A1 (FOXA1) is a pivotal transcription factor involved in cell proliferation, apoptosis, and differentiation, organogenesis, and tumor progression [31, 32]. It is required for the chromatin recruitment of estrogen receptor that regulates chromatin remodeling, estrogen receptor-related gene expressions, and tumor cell proliferation [33, 34]. It has been shown that there was an inverse correlation between the levels of *miR-132* and FOXA1 expressions. *MiR-132* reduced the breast tumor cells proliferation via FOXA1 targeting [35]. There was also *miR-132* down regulation in thyroid tumor tissues and cell lines. It reduced thyroid tumor cell proliferation and invasion by *FOXA1* inhibition [36]. Cisplatin (CDPP) is one of the main therapeutic drugs in nasopharyngeal carcinoma (NPC), however there is a noticeable ratio of resistance among the patients [37]. It has been reported that there was *miR-132* down regulation in NPC patients. It also induced CDDP sensitivity in NPC cells through *FOXA1* suppression [32]. Long non-coding RNAs (lncRNAs) are a family of the ncRNAs that regulate cell growth and tumorigenesis by post-transcriptional regulation and miRNAs sponging [38]. They are involved in tumorigenesis, tissue development, embryogenesis, and inflammation [39–41]. Pseudogene belongs to the lncRNAs family that regulates the gene expression during tumor progressions. *PTTG3P* is a pseudogene that is up regulated in pancreatic ductal adenocarcinoma (PDAC) tissues. It has been observed that there were correlations between the *PTTG3P* up regulation, larger tumor size, poor prognosis, and poor differentiation in PDAC tissues. *PTTG3P* induced tumor growth and invasion through *miR-132-3p* sponging that resulted in FOXM1 targeting [42]. It has been observed that there were *LINC01551* up regulation in NPC tissues and cells. *LINC01551* induced malignant transformation of NPC by *miR-132-5p* sponging [43].

### Developmental transcription factors

SOX5 belongs to the Sox family of developmental transcription factors involved in regulation of embryogenesis, cell differentiation, proliferation, and migration [44]. There were *miR-132* down regulations in invasive pituitary tumor tissues and cell lines. It reduced cell proliferation and invasion through SOX5 suppression [45]. Circular RNAs (CircRNAs) are endogenous RNAs characterized by closed continuous loops without polyadenylated tail [46]. They are involved in different cellular mechanisms such as chromatin remodeling, cell proliferation, apoptosis, invasion, and differentiation [47, 48]. It has been observed that there was *circDOCK1* up regulation in bladder cancer (BCa) cells. *CircDOCK1* induced cell proliferation and migration by *miR‐132‐3p* sponging that resulted in SOX5 up regulation [49]. SOX2 is a developmental transcription factor that participates in self-renewal process and tumor progression [50]. *NEAT1* sponged *miR-132* to up regulate SOX2 in glioma cells [51]. Epithelial-mesenchymal transition (EMT) is a pivotal process during tumor progression in which the tumor cells lose their epithelial feature and cell–cell adhesion to gain mesenchymal feature with high migratory and invasive properties [52–54]. EMT is orchestrated by various structural factors such as CDH1 and VIM that are regulated by EMT-related transcription factors including SNAI1, SNAI2, and TWIST [55–57]. SOX4 is a developmental transcription factor with critical functions during embryogenesis and tumorigenesis. It has been shown that *miR-132* reduced osteosarcoma (OS) cell proliferation and EMT via SOX4 targeting. There was a *miR-132* down regulation in OS cell lines in comparison with normal cells. It also regulated apoptosis by BCL-2 targeting. Moreover, *miR-132* significantly inhibited OS invasion by CDH1 up regulation, while down regulation of the mesenchymal factors such as CDH2 and VIM [58]. SOX4 has a critical role in promotion of EMT process during the prostate cancer (PCa) progression [59]. There was a significant association between miR-132 down regulation, high Gleason score, and distant metastasis. *MiR-132* inhibited prostate tumor cell migration, colony formation, and TGF-b-induced EMT by *SOX4* targeting [60]. Other studies have been reported that *miR-132-3p* inhibited the lung and liver tumor cells invasions by *SOX4* targeting [61, 62]. E2F5 belongs to the E2F family of transcription factors that regulate cell cycle progression [63]. It has been observed that there were significant *miR-132* down regulation in ovarian tumor tissues and cell lines. It suppressed ovarian tumor cell proliferation and invasion via *E2F5* targeting [64]. SIRT1 is an NAD dependent deacetylase that regulates cell death in oxidative and genotoxic stresses [65, 66]. SREBP is a leucine zipper transcription factor involved in cholesterogenesis and lipogenesis [67]. It has been observed that *miR-132* reduced glioma cell proliferation by down regulations of SIRT1 and SREBP-1c [68]. CAMP-responsive element binding protein 5 (CREB5) is a zinc-finger DNA-binding protein with pivotal functions in cell proliferation and differentiation [69]. There was a significant *SNHG5* up regulation in colorectal cancer (CRC) cells. It induced CRC invasion, while inhibited apoptosis through CREB5 up regulation following the *miR-132-3p* sponging [70].

### EMT-related transcription factors

Enhancer of zeste homolog 2 (EZH2) is one of the components of Polycomb repressor complex 2 (PRC2) that is involved in DNA methylation using DNA methyl transferases (DNMTs) recruitment [71]. It has a pivotal role in epigenetic silencing by catalyzing the H3K27me3 in promoter sequences [72]. EZH2 is also up regulated by various transcription factors like SOX4 [73]. It has been reported that the SOX4/EZH2 complex induced H3K27me3 in *miR-132* promoter sequence. *MiR-132* reduced EMT process in ovarian tumor cells by CDH1 up regulation, while CDH2 and VIM down regulations. Therefore, SOX4 was suggested as the effector of *miR-132* during EMT regulation in ovarian cancer (OC) [74]. BMI-1 is a ring finger component of PRC1 complex involved in epigenetic suppression [75]. It is an epigenetic modification protein involved in CSC self-renewal, tumor progression, and metastasis [21]. There were correlations between the BMI-1 up regulation, poor prognosis, increased invasion, and radio resistance [76, 77]. It was observed that there was *miR-132* down regulation in cervical cancer. There was also a direct association between the levels of *miR-132* expressions and radiation intensity. *MiR-132* increased radio sensitivity through *BMI-1* targeting [78]. It has been shown that there was significant *miR-132* down regulation in SKOV3/CDDP cells compared with maternal SKOV3 cells. Reduced levels of *miR-132* induced the CDDP resistance in ovarian tumor cells via *BMI-1* targeting and subsequent apoptosis inhibition [79]. ZEB2 is a zinc finger transcription factor that functions as a transcriptional co-repressor via R-SMADs binding. There were significant *SOX2OT* up regulations in Non-small-cell lung carcinoma (NSCLC) tissues and cell lines. *SOX2OT* silencing significantly reduced cell proliferation, invasion, and EMT process by *miR-132* sponging that resulted in ZEB2 up regulation [80]. There was also a significant *miR-132* down-regulation in metastatic CRC tissues in comparison with non-metastatic tumor tissues. It reduced the CRC invasion and EMT process via ZEB2 targeting. The levels of *miR-132* expressions were inversely correlated with stage, tumor size, survival, and distant metastasis in CRC patients [81].

## Structural factors

USP9X belongs to the ubiquitin-specific peptidase (USP) family involved in various cellular processes via deubiquitinaton and stabilization of target proteins. USP9X up regulation is associated with tumor cell proliferation, drug resistance, and invasion [82]. It also deubiquitinates the MCL1 as an anti-apoptotic factor to suppress cell death in NSCLC [83]. It has been reported that *miR-132* reduced NSCLC invasion via *USP9X* targeting [84]. USP22 belongs to the deubiquitinating enzyme (DUB) family of proteins involved in tumor relapse and progression [85]. USP22 silencing inhibits the tumor cell proliferation [76]. It has been reported that *SNHG16* induced colorectal tumor cell proliferation and invasion through *miR-132-3p* sponging and subsequent USP22 up regulation [86]. HN1 promotes the ubiquitin-related degradation of b-catenin that results in loss of CDH1 interaction, actin organization, and cell migration [87]. It has been reported that there was *miR-132* down regulation in breast cancer (BC) tissues in comparison with normal margins. *MiR-132* significantly inhibited BC cell proliferation and metastasis through HN1 targeting. There was also a direct association between the levels of *HN1* expression and poor survival in BC patients [23].

Derlin1 belongs to the derlin protein family that participates in endoplasmic reticulum (ER)-related degradation of misfolded proteins. It mediates retro translocation of misfolded proteins from ER to cytoplasm for the proteasomal degradation. Myocardial infarction associated transcript (MIAT) is an lncRNA associated with various human disorders such as diabetes and cancer [88]. There were significant *MIAT* up regulations in CRC tissues and cells. Silencing of *MIAT* promoted apoptosis, while suppressed CRC invasion. MIAT induced CRC cell proliferation and invasion through *miR-132* sponging that resulted in Derlin-1 up regulation [89]. Tumor suppressor candidate 3 (TUSC3) is a component of the oligosaccharyl transferase complex involved in regulation of the N-linked protein glycosylation. It is a tumor suppressor frequently down regulated in different cancers. It has been reported that *miR-132* promoted temozolomide resistance and glioblastoma initiating cells (GICs) phenotype formation by TUSC3 targeting in glioblastoma (GBM). TUSC3 also significantly down regulated the STAT3 and MDM2, while up regulate p53 [90].

TTK is a pivotal dual specificity kinase during mitotic checkpoint, centrosome duplication, and chromosome stability [91]. It induces cell proliferation and migration via AKT activation [92]. HLF is a transcription factor involved in resistance toward oxidative stress-induced apoptosis [93]. It has been reported that there were *miR-132* down regulations in glioma tissues and cell lines that were associated with advanced tumor grades. HLF-mediated *miR-132* inhibited glioma cell invasion and radio resistance via TTK inhibition [94]. P21-activated kinase 1 (Pak1) is a serine/threonine kinase that has key functions in cell migration, apoptosis, and neoplastic transformation [95, 96]. It regulates various cellular processes such as tumor cell invasion, drug resistance, angiogenesis, and EMT [97]. It exerts its oncogenic function by preventing apoptosis using different cascades including FOXO1, CLL/BCL-2, or DLC1 [98, 99]. ATF2 belongs to the b-ZIP family of transcription factors that regulates cellular differentiation and survival [100]. FN1 is an extracellular matrix glycoprotein involved in angiogenesis and tumor cell invasion [101]. It has been observed that *miR-132* affected the hematogenous metastasis in GC. PAK1 down regulated the *miR-132* via phosphorylation of ATF2 that prevents ATF2 to enter to the nucleus where it functions as an inducer of *miR-132* expression. *MiR-132* also reduced the levels of CD44 and FN1 expressions to promote lymphocytic mediated apoptosis of tumor cells. There were significant *miR-132* down regulations in GC tissues that were associated with hematogenous metastasis. ATF2 up regulated the *miR-132* that subsequently regulated the CD44/FN1/SIRT1/BDNF axis to recruit lymphocytes to suppress hematogenous metastasis in GC [102]. Receptor tyrosine kinases (RTKs) are the cell surface receptors for many extracellular signals such as hormones and growth factors. Aberrant RTK activation is implicated in progression of different tumors [103, 104]. MUC13 is a trans-membrane mucin associated with abnormal cell proliferation and tumor growth [105]. It activates the HER2, ERK, and AKT, while suppresses p53 expression [106]. It has been reported that there was a significant *MUC13* up regulation in GC tissues in comparison with normal margins. *MiR-132-3p* suppressed GC progression by *MUC13* targeting that resulted in activation of HER2 signaling [107].

Glucose transporter 1 (GLUT1) is a glucose uniporter across the erythrocytes plasma membranes. It has been shown that there was significant *miR-132* down regulation in prostate tumor cells. *MiR-132* silencing promoted the cell proliferation by induced glycolysis following the GLUT1 up regulation [108]. PEA-15 is an anti-apoptotic factor involved in TRAIL resistance of tumor cells. PEA15 over expression has been reported in GBM, leukemia, and NSCLC patients who were resistant against TRAIL [109–111]. *MiR-132* reduced tumor cell proliferation and invasion, while increased apoptosis by targeting PEA-15 in astrocytoma. It was also observed that the *miR-132* was regulated by CREB and KLF transcription factors [112]. Cyclin E1 (CCNE1) belongs to the cyclin family of proteins that regulates cyclin-dependent kinase 2 (CDK2) during cell cycle G1/S transition. It has been observed that there were *miR-132* down regulations in OS tissues compared with normal bone tissues. *MiR-132* reduced OS cell proliferation, colony formation, and in vivo growth via CCNE1 targeting [113].

## PI3K/AKT pathway

**Fig. 1:**
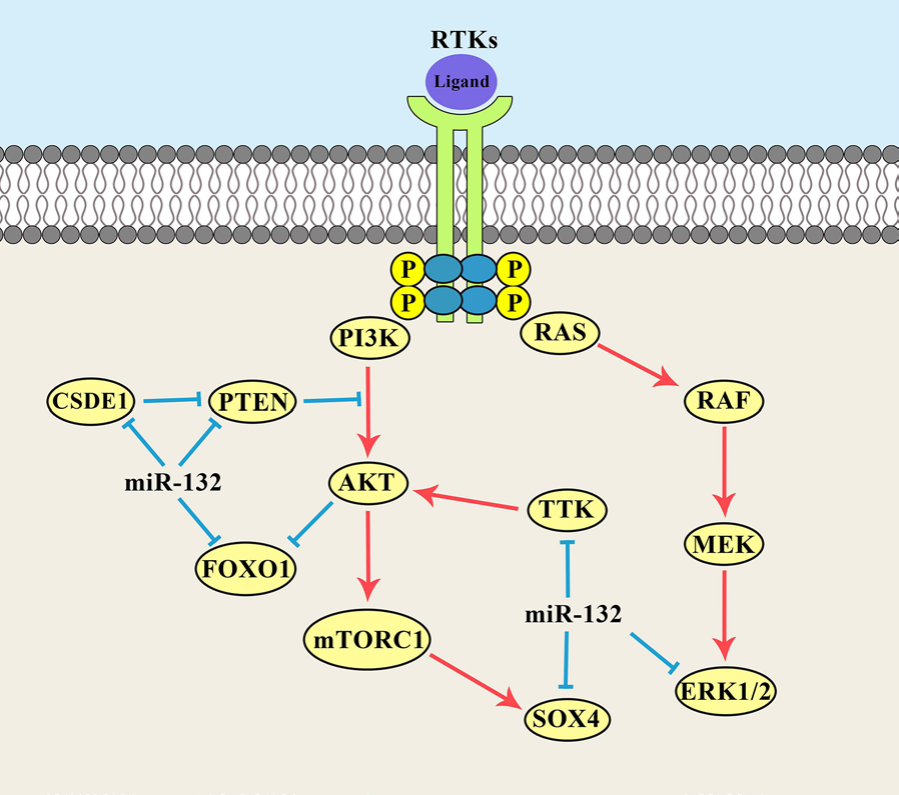
Molecular mechanisms of miR-132 in regulation of PI3K/AKT and MAPK and signaling pathways during tumor progression

The PI3K/AKT is an important signaling pathway that transfers the extracellular signals such as growth factors and hormones into the cells to regulate cell proliferation, metabolism, and apoptosis. PI3K activation by the RTKs and G-protein coupled receptors (GPCRs) subsequently phosphorylates and activates the AKT (Fig. 1). AKT is a serine/threonine kinase that has various effectors including CREB, FOXO, and mTOR [114, 115]. FOXO1 phosphorylation by AKT results in nuclear export and proteasome-dependent degradation [116]. It is a transcriptional regulator of apoptosis and CDK inhibitors such as BIM, FASL, p27, and p21 that inhibit G1/S transition and promote apoptosis [117, 118]. It has been reported that there was a significant *miR-132* up regulation in laryngeal squamous cell carcinoma (LSCC) cells. *MiR-132* promoted LSCC cell proliferation and tumor growth by PI3K/AKT activation and FOXO1 targeting [119]. PTEN as a tyrosine phosphatase inhibits the PI3K/AKT signaling by PIP3 dephosphorylation that results in AKT inhibition [120]. Therefore, PTEN down regulation activates the AKT/ERK pathway to regulate tumor cell proliferation and invasion. PTEN up regulation also promotes tumor cells apoptosis [121]. Moreover, it is a potent regulator of EMT progression [122]. It has been reported that there was *miR-132* up regulation in pancreatic carcinoma that was associated with poor prognosis. *MiR-132* reduced cell invasion and proliferation of pancreatic tumor cells through *PTEN* targeting [123]. *MiR-132* increased doxorubicin resistance of BC cells through *PTEN* targeting [124]. Cold shock domain containing E1 (CSDE1) is an RNA binding protein (RBP) that is involved in tumor progression [125]. It has been observed that CSDE1 reduced thyroid tumor cell proliferation. CSDE1 down regulated the PTEN that resulted in AKT activation. *MiR-132* also targeted the CSDE1 in thyroid tumor cells [126]. LAPTM4B is an inducer of tumor cell proliferation, invasion, and drug resistant by activation of PI3K/AKT pathway [127]. There were correlations between *miR‐132‐3p* down regulation, TNM staging, and tumor relapse in BC patients in which the patients with stage II/III had lower levels of *miR-132-3p* expressions compared with patients with stage I, and patients with recurrence had significantly lower levels of *miR-132-3p* expression. *MiR‐132‐3p* suppressed the breast tumor cell proliferation and invasion through LAPTM4B inhibition that resulted in inhibition of the PI3K/AKT/mTOR axis [128]. PIK3R3 is the regulatory subunit of the PI3K that phosphorylates phosphatidylinositol as a second messenger in intracellular signal transductions. It binds to the activated tyrosine kinases by SH2 domains to exert its functions. It has been reported that the *LINC00160* knock down reduced the levels of PIK3R3 through *miR-132* up regulation that resulted in reduced hepatocellular carcinoma (HCC) tumor cell drug resistance. There were also *LINC00160* and *PIK3R3* up regulations in HCC tissues. *LINC00160* sponged the *miR-132* to up regulate PIK3R3. *LINC00160* silencing inhibited the HCC cell autophagy and proliferation, while induced apoptosis through PIK3R3 and ATG5 down regulations via promotion of miR-132 [129].

## TGF-β pathway

Transforming growth factor b (TGF-β) is a secreted multi-faceted cytokine involved in regulation of embryogenesis, apoptosis, inflammation, and tissue homeostasis using SMAD family of transducer proteins. It triggers and maintains the EMT process by promotion of signaling pathways and transcription factors. Both SMAD-dependent and independent cascades are recruited by TGFβ to induce EMT during tumor progression. It has been reported that there was *miR-132* down regulation in cervical cancer samples. *MiR-132* reduced cervical tumor cell growth and invasion by *SMAD2* targeting that resulted in EMT and cell cycle regulations. *MiR-132* silencing promoted EMT via CDH1 down regulation, while VIM, FN1, SNAI1, SNAI2, and TWIST2 up regulations [130]. A significant *miR-132* down regulation was also observed in BC tissues with metastatic lymph nodes. *MiR-132* silencing promoted the breast tumor cell invasion and increased the levels of EMT-related markers and TGFβ1/SMAD2 expressions. There was an inverse association between *SMAD2* and *miR-132* expression levels in BC tissues. *MiR-132* inhibited the EMT by down regulations of CDH2, ZEB1, SNAI1, and VIM in BC cells. It regulated the EMT process through TGFβ1/SMAD2 signaling pathway [131]. It has been observed that there was *ILF3-AS1* up regulation in retinoblastoma (RB) tissues compared with normal controls. Levels of *ILF3-AS1* expressions were directly correlated with advanced stage and optic nerve metastasis. *ILF3-AS1* silencing significantly decreased malignant behaviors and in vivo tumor growth. *ILF3-AS1* promoted RB progression through *miR-132–3p* sponging that up regulated the *SMAD2* [132]. *MiR-132* was reported to increase cisplatin sensitivity in Oral squamous cell carcinoma (OSCC) cells. There was also significant *TGFβ1* up regulation in OSCC tissues that was conversely associated with *miR-132* expression. *MiR-132* also reduced OSCC cell proliferation and invasion by targeting the TGFβ1/SMAD2-3 axis [133]. Glucocorticoids are a class of corticosteroids with therapeutic values in lymphoid cancer, however some of the patients are insensitive to this treatment option [134]. Dexamethasone (DEX) is a glucocorticoid medication of tumor progression that promotes EMT and self-renewal via activation of the JNK and TGFβ pathways [135]. It has been observed that the DEX was involved in regulation of *miR-132* promoter methylation. *MiR-132* increased pancreatic tumor cell clonogenicity and EMT through TGFβ regulation [136].

## Other signaling pathways

Mitogen-activated protein kinase (MAPK) signaling pathway is categorized to the ERK, JNK, and p38 cascades in mammalian cells which are involved in regulation of stress responses, cell proliferation, and differentiation. This signaling pathway transmits the extracellular signals via a sequential activation of MAP4K, MAP3K, and MAPKAPK. JNK and p38 are mainly activated in stress response, while the ERK1/2 are associated with cell proliferation and differentiation [137]. ERK1 is involved in tumor relapse, invasion, and drug resistance [138]. It can be regulated by the *miR-132* during tumor progressions (Fig. 1). *MiR-132* suppressed CRC cell proliferation and Adriamycin (ADM) resistance, while promoted apoptosis through ERK1 targeting [139]. There were *XIST* up regulations in CRC tissues and cells that were directly associated with TNM stage and tumor size. *XIST* induced colorectal tumor cell proliferation via the miR-132-3p/ERK2 axis [140]. Hedgehog (Hh) is a developmental signaling pathway involved in cell differentiation and embryogenesis. It is activated by Hh ligands binding with PTCH receptor that results in activation of GLI transcription factors [141]. Aberrant Shh activation induces the cell proliferation by Myc, PTCH, and CCND1 up regulations [142, 143]. It has been reported that *miR-132* increased pancreatic tumor cell proliferation via Hh pathway [144]. Hippo signaling is involved in regulation of organ volume by the maintenance of cell proliferation/apoptosis balance [145, 146]. Yes-associated protein (YAP) is one of the key effectors of Hippo signaling pathway which has a pivotal function in induction of cell proliferation and invasion, while apoptosis suppression. It has been reported that *miR-132* induced hepatoma cell apoptosis, while suppressed their proliferation and invasion through YAP targeting [147].

### Conclusions

In present review we summarized all of the studies that have been evaluated the role of miR-132 in different cancers. This review clarifies the cell and molecular mechanisms that are regulated by miR-132 during tumor progressions. It has been reported that the miR-132 mainly functions as a tumor suppressor; it has also oncogenic functions especially in pancreatic tumors. It mainly exerts its roles during tumor progressions by regulation of the transcription factors and signaling pathways. Present review clarifies the tumor specific molecular mechanisms of miR-132 to introduce that as an efficient non-invasive diagnostic marker in various cancers.

## Data Availability

The datasets used and/or analyzed during the current study are available from the corresponding author on reasonable request.

## Abbreviations

miRNAs: MicroRNAs
ncRNAs: Non-coding RNAs
lncRNAs: Long noncoding RNAs
siRNAs: Small interfering RNAs
circRNA: Circular RNA
CSC: Cancer stem cells
Fox: Forkhead box proteins
FOXA1: Forkhead box protein A1
NPC: Nasopharyngeal carcinoma
BCa: Bladder cancer
EMT: Epithelial-mesenchymal transition
OS: Osteosarcoma
PCa: Prostate cancer
CREB5: CAMP-responsive element binding protein 5
CRC: Colorectal cancer
EZH2: Enhancer of zeste homolog 2
PRC2: Polycomb repressor complex 2
DNMTs: DNA methyl transferases
OC: Ovarian cancer
NSCLC: Non-small-cell lung carcinoma
USP: Ubiquitin-specific peptidase
MIAT: Myocardial infarction associated transcript
TUSC3: Tumor suppressor candidate 3
GICs: Glioblastoma initiating cells
GBM: Glioblastoma
Pak1: P21-activated kinase 1
GLUT1: Glucose transporter 1
CCNE1: Cyclin E1
CDK2: Cyclin-dependent kinase 2
GPCRs: G-protein coupled receptors
LSCC: Laryngeal squamous cell carcinoma
CSDE1: Cold shock domain containing E1
RBP: RNA binding protein
HCC: Hepatocellular carcinoma
TGF-β: Transforming growth factor b
RB: Retinoblastoma
OSCC: Oral squamous cell carcinoma
DEX: Dexamethasone
MAPK: Mitogen-activated protein kinase
Hh: Hedgehog
YAP: Yes-associated protein
CDPP: Cisplatin
ADM: Adriamycin
